# The Role of Vitamin E in Preventing and Treating Osteoarthritis – A Review of the Current Evidence

**DOI:** 10.3389/fphar.2018.00946

**Published:** 2018-08-21

**Authors:** Kok-Yong Chin, Soelaiman Ima-Nirwana

**Affiliations:** Department of Pharmacology, Faculty of Medicine, Universiti Kebangsaan Malaysia Medical Centre, Kuala Lumpur, Malaysia

**Keywords:** cartilage, chondrocytes, oxidative stress, tocopherol, tocotrienol

## Abstract

Osteoarthritis is a debilitating disease of the joint involving cartilage degeneration and chondrocytes apoptosis. Oxidative stress is one of the many proposed mechanisms underpinning joint degeneration in osteoarthritis. The current pharmacotherapies emphasize pain and symptomatic management of the patients but do not alter the biological processes underlying the cartilage degeneration. Vitamin E is a potential agent to prevent or treat osteoarthritis due to its antioxidant and anti-inflammatory effects. This review aims to summarize the current evidence on the relationship between vitamin E and osteoarthritis derived from preclinical and human studies. Cellular studies showed that vitamin E mitigated oxidative stress in cartilage explants or chondrocyte culture invoked by mechanical stress or free radicals. Animal studies suggested that vitamin E treatment prevented cartilage degeneration and improve oxidative status in animal models of osteoarthritis. Low circulating or synovial vitamin E was observed in human osteoarthritic patients compared to healthy controls. Observational studies also demonstrated that vitamin E was related to induction or progression of osteoarthritis in the general population. Vitamin E supplementation might improve the outcomes in patients with osteoarthritis, but negative results were also reported. Different isomers of vitamin E might possess distinct anti-osteoarthritic effects. As a conclusion, vitamin E may retard the progression of osteoarthritis by ameliorating oxidative stress and inflammation of the joint. Further studies are warranted to develop vitamin E as an anti-osteoarthritis agent to reduce the global burden of this disease.

## Introduction

Free radicals are involved in many physiological functions of chondrocytes, such as intracellular signaling, apoptosis, cytokine production and extracellular matrix remodeling (Lepetsos and Papavassiliou, 2016). However, increased oxidative stress due to the imbalance of antioxidants and oxidants can be harmful to the chondrocytes (Lepetsos and Papavassiliou, 2016). Apart from damaging the cellular macromolecules, free radicals can also activate the nuclear factor kappa-light-chain-enhancer of activated B cell (NFκB), phosphoinositide 3-kinase (PI3K) and c-Jun N-terminal kinase (JNK) pathways, which result in the senescence and apoptosis of chondrocytes. Consequently, cartilage remodeling will be impaired, giving rise to cartilage thinning and osteoarthritis. This is evidenced by the low levels of antioxidants, high levels of oxidants and oxidized/nitrated adducts of macromolecules in the synovial fluid of osteoarthritic patients compared to healthy controls (Regan et al., 2008; Ahmed et al., 2016). A higher level of F(2)-isoprostane, a stable marker of oxidative stress *in vivo*, has been found in patients with rheumatic diseases, including osteoarthritis (Basu et al., 2001). High level of nitric oxide, a nitrogen reactive species produced by the inducible nitric oxide synthase at the joint, is also responsible for increased pain signaling among osteoarthritic patients (Hancock and Riegger-Krugh, 2008). Therefore, antioxidant therapy represents a potential avenue to tackle the degenerative joint changes and pain in osteoarthritis.

Vitamin E is a strong antioxidant found in plants. Structurally, it consists of a chromanol ring and an isoprenoid side chain. Vitamin E is a broad term referring to tocopherols and tocotrienols, which can be further divided into alpha-, beta-, gamma-, and delta-isomers based on the position of side chains on the chromanol ring (Aggarwal et al., 2010; Colombo, 2010). Alpha-tocopherol is the most prevailing vitamin E isomer in nature and in the body (Chin and Ima-Nirwana, 2014). It is present in the synovial fluid despite being an aqueous environment (Sutipornpalangkul et al., 2009; Angthong et al., 2013; Suantawee et al., 2013). The antioxidant activity of vitamin E relies on the hydroxyl group on the chromanol ring, which readily donates hydrogen to reduce the free radicals (Peh et al., 2016). *In vitro* and *in vivo* studies have demonstrated that the antioxidant activity of tocotrienol is superior to tocopherol due to three reasons: (1) tocotrienol is more uniformly distributed across the lipid membrane; (2) tocotrienol, with the double bonds on its isoprenoid side chain, offers more interaction with free radicals; (3) tocotrienol has a higher redox cycling efficiency compared to tocopherol (Packer et al., 2001). Apart from acting as a free radical scavenger, vitamin E can modulate nuclear factor-erythroid 2-related factor 2 (NRF2), a transcription factor regulating the expression of antioxidant enzymes (Dworski et al., 2011). Vitamin E supplementation has been shown to upregulate the activity of antioxidant in the musculoskeletal system (Chin et al., 2013). It can also inhibit NFκB pathway and inflammation (Elisia and Kitts, 2015), the key factors sustaining the vicious cycle of joint destruction in osteoarthritis (Houard et al., 2013). These properties suggest that vitamin E could be used as an agent to suppress oxidative stress and inflammation underlying the pathogenesis of osteoarthritis.

Currently, the pharmacotherapeutic approach for osteoarthritis aims to relieve the pain and symptoms experienced by the patients using paracetamol, non-steroidal anti-inflammatory drugs and opioids (Hochberg et al., 2012). Recently, biologics have been developed to address the biological changes of osteoarthritis, but they are still in the experimental stage (Chevalier et al., 2013). Vitamin E, in the form of supplement or dietary intervention, may retard the progression of osteoarthritis. It could reduce the morbidity of osteoarthritis, which ranks as the 11th highest contributor to the global disability (Cross et al., 2014). It could potentially benefit the 3.8% population worldwide suffering from knee osteoarthritis, and the 0.85% suffering from hip osteoarthritis (Cross et al., 2014). Therefore, this review aims to summarize the current evidence on the relationship between vitamin E and osteoarthritis derived from preclinical and human studies.

## Literature Search

The literature search was performed via Pubmed and Scopus databases using the keywords (“vitamin E” OR “tocopherol” OR “tocotrienol”) AND (“osteoarthritis” OR “cartilage” OR “chondrocytes”). The search was conducted in April 2018. Only original research articles in English published after 1995 were included. Both authors decided the articles to be included in this review.

It is noted that the term “vitamin E” was used indiscriminately to refer to alpha-tocopherol in most studies. Therefore, in the discussion below, we adopted the term used in the original papers, which usually refers to alpha-tocopherol, unless stated otherwise.

## Preclinical Evidence from Cellular Studies

In a model of leporine cartilage explants, Tiku et al. (2000) investigated the effects of oxidative stress and antioxidants on cartilage matrix degradation (Tiku et al., 2000). When the explants were treated with hydrogen peroxide and calcium ionophore, the cartilage matrix degraded, releasing proline, the major component of collagenous polypeptides, which was then detected by the researchers (Tiku et al., 2000). Pretreatment with superoxide dismutase (100 units/ml) and catalase (1000 units/ml) did not prevent the chondrocyte-mediated matrix degradation, maybe because the molecules were too large to penetrate the explants (Tiku et al., 2000). On the other hand, vitamin E at 250 and 500 mM prevented matrix degradation more effectively than vitamin C, indicating that the process may be mediated by lipid peroxidation (Tiku et al., 2000). This was evidenced by the observations that vitamin E also lowered malondialdehyde and hydroxynonenal-protein adducts from chondrocytes (Tiku et al., 2000). Other inhibitors of lipid peroxidation, like butylated hydroxytoluene and deferoxamine, also prevented matrix degradation in the explants in a manner similar with vitamin E (Tiku et al., 2000).

Since cartilage damage is usually initiated with mechanical stress, Beecher et al. (2007) introduced mechanical stress on human cartilage explants and tested the protective effects of several antioxidants (Beecher et al., 2007). Extensive chondrocyte apoptosis and DNA fragmentation were observed at the cartilage explants induced with mechanical stress (Beecher et al., 2007). The viability of chondrocytes was reduced to 40% in the superficial layer and 55% in the middle zone (Beecher et al., 2007). Vitamin E at 100 μM, along with other antioxidants, such as *N*-acetylcysteine at 2.5 mM and superoxide dismutase at 50 μM, prevented these adverse changes (Beecher et al., 2007). However, this study did not examine the molecular mechanism responsible for protective effects of these antioxidants. The authors hypothesized that apart from the apparent extinguishment of oxidative and nitrative stress, mitogen kinase pathway might be implicated (Beecher et al., 2007).

Using primary rat chondrocytes from articular cartilage, Bhatti et al. (2013) demonstrated that vitamin E pretreatment (50 and 100 μM) preserved the proteoglycan content in the extracellular matrix of the culture (Bhatti et al., 2013). It also upregulated the expression of genes coding for aggrecan (Agc1), collagen type II alpha 1 (Col2a1) and proliferating cell nuclear antigen (PCNA), and downregulated the expression of collagen type I alpha 1 (Col1a1) and caspase 3 (Casp3) (Bhatti et al., 2013). Therefore, the differentiation index of chondrocytes calculated as a ratio of Col2a1: Col1a1 increased with treatment (Bhatti et al., 2013). Since Col2a1 coded the protein for hyaline cartilage, a high differentiation index indicated that the hyaline phenotype of chondrocytes was maintained (Marlovits et al., 2004). Cell viability also increased with vitamin E treatment, in conjunction with a reduction in nitrite level (an indicator of nitritive stress) (Bhatti et al., 2013). In preventing apoptosis and senescence of chondrocytes, vitamin E at 100 μM was more effective than 50 μM (Bhatti et al., 2013).

Only one *in vitro* study observed a negligible effect of alpha-tocopherol in synoviocytes experiencing oxidative stress induced by hypoxanthine-xanthine oxidase (HX/XO) which generated superoxide anions (Galleron et al., 1999). Alpha-tocopherol (10^−5^ to 10^−3^ M) did not protect the cells from mitochondrial injury, DNA fragmentation and apoptosis induced by superoxide anions (Galleron et al., 1999). The authors postulated that alpha-tocopherol molecules were converted to alkoxyl radicals by superoxide anions, but there were no other antioxidants to reduce them (Galleron et al., 1999). Other researchers suggest that vitamin E will act as prooxidant rather than antioxidant in this circumstance (Chin and Ima-Nirwana, 2014). Considering all evidence, vitamin E may help to ameliorate oxidative stress harmful to chondrocytes at lower concentration but increase oxidative damage at higher concentration.

## Preclinical Evidence From Animal Studies

The importance of vitamin E in joint health was hinted by a preliminary observational study comparing the synovial vitamin E level between dogs with osteoarthritis caused by spontaneous cranial cruciate ligament rupture (*n* = 6) and healthy dogs (*n* = 6) (de Oliveira El-Warrak et al., 2012). They found that dogs with osteoarthritis had a significantly higher vitamin E level compared to healthy dogs despite the lack of difference in vitamin A, selenium ad L-lactate (de Oliveira El-Warrak et al., 2012). The authors postulated that there was increased mobilization of vitamin E to the damaged joint to combat the elevated oxidative stress (de Oliveira El-Warrak et al., 2012). However, the dogs studied were of heterogenous breed and the sample size of the study was too small to reach a conclusion. In the subsequent study, Rhouma et al. (2013) treated dogs with osteoarthritis induced by cranial cruciate ligament transection (causing joint instability) with oral vitamin E 400 IU/day for 55 days (Rhouma et al., 2013). They revealed that the supplementation increased the serum and synovial vitamin E (Rhouma et al., 2013). This corroborated with a significant improvement in pain determined by visual analog scale (assessed by a technician; significant at day 55) and electrodermal activity (significant at day 28) compared to the control group (Rhouma et al., 2013). The score of cartilage lesion at the lateral femoral condyles, synovial nitrite oxide and prostaglandins were also lower in the treatment group compared to the control (Rhouma et al., 2013).

Similarly, the effects of vitamin E have also been examined in rodent models of osteoarthritis induced by monosodium iodoacetate or joint instability due to surgery. Heidar et al. (2014) administered vitamin E (600 mg/kg for three times per week) in rats with osteoarthritis induced by monosodium iodoacetate (Heidar et al., 2014), which caused the death of chondrocytes by inhibiting glycolysis, resulting in cartilage breakdown (Jiang et al., 2013). Vitamin E was found to decrease the serum tumor necrosis factor alpha, interleukin-6 levels and superoxide dismutase activity in the rats (Heidar et al., 2014). Scanning electron microscope examination of the cartilage showed less damage on the territorial matrix and collagen fibrin sheet (Heidar et al., 2014). In another study using osteoarthritis model induced by anterior cruciate ligament transection concurrent with medial meniscectomy, the effects of intra-articular hyaluronic acid (25 mg/2.5 ml), tenoxicam (a NSAID; 20 mg/2 ml) and vitamin E (300 mg/2 ml alpha-tocopherol) individually once a week for 5 weeks were compared (Ozkan et al., 2015). Ozkan et al. (2015) showed that all treatments, including vitamin E, improved the joint histological score graded by Mankin system (Ozkan et al., 2015). When individual components of the scores were scrutinized, vitamin E significantly enhanced the proteoglycan content of the cartilage, but not structure and cellular improvements (Ozkan et al., 2015). In comparison, tenoxicam and hyaluronic acid performed slightly better than vitamin E in all aspects (Ozkan et al., 2015).

Kurz et al. (2002) determined the effects of a diet enriched with vitamin E, C, A, B6, B2, and selenium on STR/1N mice, which naturally experienced osteoarthritis due to varus-deformity-induced mechanical overload of the medial tibial plateau, as well as normal Balb/C mice (Kurz et al., 2002). The special diet decreased osteoarthritic lesion at the joints and increased serum glutathione peroxidase activity in both strains of mice (Kurz et al., 2002). In STR/N1 rats, the proportion of mice with grade 4 lesion (most severe) decreased from 81 to 18%, while in normal Balb/C mice, the proportion reduced from 21 to 14% (Kurz et al., 2002). Immunostaining results demonstrated that protein expression of glutathione peroxidase and copper/zinc-superoxide dismutase at the articular cartilage, and glutathione peroxidase at the synovium of both mice strains were increased following the treatment (Kurz et al., 2002). In addition, the manganese and copper/zinc superoxide dismutase expression only increased at the synovium of STR/N1 mice (Kurz et al., 2002). However, because the vitamins were administered together, the individual effects of vitamin E could not be delineated.

In a recent study, Bhatti et al. (2017) described the effects of alpha-tocopherol in counteracting oxidative stress induced by mesenchymal stem cell transplantation in rats with osteoarthritis induced by anterior cruciate ligament transection with meniscectomy (Bhatti et al., 2017). Pretreatment of mesenchymal stem cells with alpha-tocopherol resulted in better homing, formation of hyaline cartilage with good surface regularity, thickness, as well as integration with native tissue compared to untreated mesenchymal stem cells (Bhatti et al., 2017). As a result, the knee joint treated with mesenchymal stem cells with alpha-tocopherol had a better histological score, higher expression of aggrecan (Acan) and Col2a1 (Bhatti et al., 2017). The treated knee also expressed reduced gene expression of Casp3, vascular endothelial growth factor (VEGF) and Col1a1, as well as increased PCNA, TGFβ, Acan and Col2a1 (Bhatti et al., 2017). Changes in the knee treated with mesenchymal stem cells incubated with vitamin C were relatively modest, without improvement in Casp3 and VEGF as observed in the vitamin E-treated group (Bhatti et al., 2017).

The preclinical evidence suggests that vitamin may prevent joint degeneration and improve function in animals. This may be achieved by halting the vicious cycle of cartilage degradation by suppressing oxidative stress and inflammation induced by mechanical stress.

## The Relationship Between Vitamin E and Osteoarthritis In Human Studies

The following discourse is arranged according to the relationship between vitamin E and joint health in humans, i.e., positive (higher vitamin E intake/level, better joint health), negligible (vitamin E is not associated with joint health) and negative effects (higher vitamin intake/level, worse joint health). The heterogeneity of the findings suggests a U-shape relationship between vitamin E and joint health.

In the discussion, the term “effect” was avoided unless the study design can infer causality. In each section, the studies were arranged according to the level of evidence, starting from case-control, cross-sectional and prospective studies, to clinical trials.

### Positive Relationship Between Vitamin E and Joint Health

The importance of vitamin E in protecting cartilage health has been illustrated in several case-control studies. Surapaneni and Venkataramana (2007) showed that among Indian subjects (aged 35–60 years), the osteoarthritic patients (*n* = 20) had lower circulating vitamin E and vitamin C, in conjunction with lower erythrocytic glutathione level and catalase activity and increased erythrocytic malondialdehyde level, glutathione transferase and peroxidase activity than healthy control (*n* = 20) (Surapaneni and Venkataramana, 2007). Bhattacharya et al. (2012) also obtained similar observation in their osteoarthritic patients (*n* = 40, aged 40–70 years), alongside increased ceruloplasmin, C-reactive protein and interleukin-6 (Bhattacharya et al., 2012). However, the oxidative status of the blood might not reflect the condition in the joint space. This was best illustrated by the study of Suantawee et al. (2013). They found that osteoarthritic patients with Kellgren–Lawrence scale 3–4 (mean age = 69.2 ± 1 years) had significantly higher plasma malondialdehyde and nitrite level, as well as lower vitamin E, Trolox equivalent antioxidant capacity (TEAC), and ferric reducing antioxidant power (FRAP) than healthy control (*n* = 35, mean age = 68.6 ± 1.2 years) (Suantawee et al., 2013). In the osteoarthritic patients, synovial fluid was sampled to test the correlation between circulating and synovial markers of oxidative stress. Only FRAP showed a significant correlation, but not vitamin E, malondialdehyde and TEAC (Suantawee et al., 2013), highlighting the discordance between vitamin E level and oxidative status in the circulation and in the joint space.

Studies on vitamin E level in the joint space of patients with osteoarthritis are relatively limited. Sutipornpalangkul et al. (2009) demonstrated that synovial vitamin E level but not circulating vitamin E level was lower in Thai patients with primary osteoarthritis (*n* = 32, aged 55–88 years) compared to patients with knee joint injury but without osteoarthritis (*n* = 10, aged 19–42 years) (Sutipornpalangkul et al., 2009). The other synovial markers of oxidative stress (thiobarbituric acid-reactive-substance, iron, glutathione, superoxide dismutase, and glutathione peroxidase) were similar between these groups (Sutipornpalangkul et al., 2009). This was supported by the study of Angthong et al. (2013), whereby synovial vitamin E level was significantly lower in patients with severe osteoarthritis (*n* = 9, with Knee Society Score ≤ 46) compared to patients with mild-moderate osteoarthritis (*n* = 14, with Knee Society Score > 46) (Angthong et al., 2013). A significant inverse correlation was also detected between Knee Society Score and vitamin E level, but not with other oxidative stress markers (iron, glutathione, and thiobarbituric acid-reactive-substance) (Angthong et al., 2013). The studies mentioned above only involved a limited number of subjects. Although the term vitamin E was used, often it referred to alpha-tocopherol only. The presence of other vitamin E isomers in synovial fluid remains uncertain.

In the Johnston County Osteoarthritis Project, the relationship between serum isoforms of tocopherol and knee osteoarthritis among 400 subjects was examined (200 osteoarthritic patients with Kellgren–Lawrence scale ≥ 2 and 200 healthy controls with Kellgren–Lawrence scale = 0; age 45–92 years) (Jordan et al., 2004). The study showed that alpha-tocopherol was negatively associated with knee osteoarthritis in men (Odds ratio (OR): 0.1, 95% confidence interval (CI): 0.01–1.3; highest tertile versus lowest tertile) but not in women (Jordan et al., 2004). However, the association between gamma-tocopherol and osteoarthritis was positive, which complicated the interpretation of this study (Jordan et al., 2004). This would be discussed in the following section. The cross-sectional study by Seki et al. (2010) considered the relationship between various circulating tocopherol isomers and osteoarthritis among 562 Japanese subjects aged ≥40 years (Seki et al., 2010). The osteoarthritic patients (with Kellgren–Lawrence scale ≥ 2) had lower beta/gamma-tocopherol level compared to healthy controls (Seki et al., 2010). Logistic regression showed that subjects at the highest tertile of beta/gamma-tocopherol were associated with lower risk for osteoarthritis (OR: 0.52, 95% CI: 0.29–0.93) (Seki et al., 2010). This result persisted after adjustment for alpha-tocopherol level (Seki et al., 2010).

Structural changes in the joint usually precede functional changes in osteoarthritis. In a cross-sectional study involving 827 Japanese rural residents aged ≥40 years (mean age = 62.9 ± 9.3 years), the relationship between vitamin E intake and osteophyte area and minimum joint space assessed through computer-aided analysis of the knee radiographs was investigated (Muraki et al., 2014). Muraki et al. (2014) found that vitamin E intake was associated with osteophyte area (β = −0.15, 95% CI: −0.29 to −0.008) but not minimum joint space width in women (Muraki et al., 2014). In men, vitamin E was associated with neither of the two variables (Muraki et al., 2014). Barker et al. (2014a) showed that circulating gamma-tocopherol was significantly higher in patients with knee osteoarthritis and vitamin D deficiency (<20 ng/ml circulating 25-hydroxyvitamin D) (Barker et al., 2014a). These patients also suffered from quadriceps muscle weakness. The gamma-tocopherol level was negatively associated with tumor necrosis factor alpha level in these patients (Barker et al., 2014a). Taken together, gamma-tocopherol might be associated with inflammatory muscle weakness in osteoarthritic patients with vitamin D deficiency (Barker et al., 2014a). However, this study was limited by its small sample size (*n* = 56) and relatively young subjects (mean age = 48 ± 1 years). Using data from a clinical trial, Oikonomidis et al. (2017) performed secondary analysis among osteoarthritic patients administered with vitamin C and vitamin E or meloxicam (Oikonomidis et al., 2017). A negative relationship was observed between synovial alpha-tocopherol and malondialdehyde ratio with Western Ontario and McMaster Universities Arthritis Index (WOMAC) score and pain visual analogs scale (Oikonomidis et al., 2017). A shift of approximately 5 mM total antioxidant capacity (TAC) expressed as a-tocopherol equivalents was demonstrated to change the degree of osteoarthritis and pain (Oikonomidis et al., 2017). However, there was no significant association between alpha-tocopherol and Kellgren–Lawrence grade (Oikonomidis et al., 2017).

Two prospective studies supported the inverse relationship between vitamin E and cartilage health. In the Framingham Osteoarthritis Cohort Study, participants were screened two times (baseline: 1983–1985; follow-up: 1992–1993) to investigate the link between nutrient intake and osteoarthritis (McAlindon et al., 1996). Progressive osteoarthritis was defined as an increase of Kellgren–Lawrence score by 1 unit on follow up. Incident osteoarthritis was defined as the changes of Kellgren–Lawrence score from <1 to >2 on follow up (McAlindon et al., 1996). McAlindon et al. (1996) revealed that vitamin E intake assessed from food frequency questionnaire at baseline was associated with decreased progression of osteoarthritis (OR: 0.42, CI: 0.19–0.94, middle versus lowest tertile) (McAlindon et al., 1996). This association was prominent in men (OR: 0.07, CI: 0.01–0.61, highest versus lowest tertile) but not in women (McAlindon et al., 1996). None of the nutrients studied was related to incident osteoarthritis (McAlindon et al., 1996). In the follow-up of Melbourne Collaborative Cohort involving subjects (*n* = 214, aged 27–75 years) with a magnetic resonance imaging of the knee in 2009–2010 period, Wang et al. (2016) indicated that higher intake of vitamin E (OR: 0.63, 95% CI: 0.41–0.96), lutein/zeaxanthin (OR: 0.58, 95% CI: 0.34–0.99), and lycopene (OR: 0.64, 95% CI: 0.44–0.95) intake assessed using food frequency questionnaire were associated with a reduced prevalence of femoral head cartilage defects (Wang et al., 2016). However, none of the nutrients studied was linked to femoral neck bone marrow lesion (Wang et al., 2016). Since the dietary data were taken 20 years prior to the outcomes, the diet of the subjects might change during this period.

In the form of supplements, vitamin E was proven to relieve the symptoms and improve the function of osteoarthritic patients. Bhattacharya et al. (2012) revealed that vitamin E supplementation at 200 IU/day in Indian osteoarthritic patients aged 50–70 years with Kellgren–Lawrence scale 3 (*n* = 40) increased the circulating erythrocytic superoxide dismutase, glutathione peroxidase and catalase activity and malondialdehyde level (Bhattacharya et al., 2012). However, this was not a placebo-controlled study. In a randomized control trial performed by Tantavisut et al. (2017), oral vitamin E at 400 IU/day or placebo was given for 2 months in patients scheduled for total knee arthroplasty (Kellgren–Lawrence scale 3–4) (Tantavisut et al., 2017). Vitamin E treatment was noted to reduce malondialdehyde, increase alpha-tocopherol level and TEAC in the blood and synovial fluid of the patients (Tantavisut et al., 2017). Besides, it reduced nitrotyrosine-stained cells in synovial tissues but exerted no effects on nitrite and FRAP level (Tantavisut et al., 2017). Vitamin E also improved all domain of WOMAC scores (pain, stiffness, and function) and Knee Society Score after 2 months (Tantavisut et al., 2017). Correlation study showed that WOMAC score was inversely associated with TEAC values in the subjects (Tantavisut et al., 2017). The authors postulated that the reduction in nitrative stress shown by nitrotyrosine staining contributed to the analgesic effects of vitamin E shown in this study. The combination of vitamin C (1 g 2 times daily) and vitamin E (100 mg 3 times daily) had also been compared against meloxicam (15 mg oral daily) treatment in 46 osteoarthritic patients (Oikonomidis et al., 2014). After 20 days, both treatment groups demonstrated similar improvements in WOMAC and pain visual analog scale (Oikonomidis et al., 2014). They also improved extension and flexion deficits in the patients, but the effect of meloxicam was limited to patients with severe-extreme osteoarthritis (Oikonomidis et al., 2014). The total antioxidant capacity of the synovial fluid was significantly enhanced in the vitamin C + vitamin E supplemented group compared to meloxicam control (Oikonomidis et al., 2014). The presence of vitamin C could improve redox recycling of vitamin E, thereby potentiate its antioxidant effects.

The clinical trial by Haflah et al. (2009) deserved a special mention. They administered either palm vitamin E (*n* = 33, 400 mg/day oral) or glucosamine sulfate (n = 31, 1.5 g/day oral) to Malay and Chinese osteoarthritic patients aged 58–59 years with Kellgren–Lawrence scale 2–3 for 6 months (Haflah et al., 2009). This was the only clinical trial conducted on a palm vitamin E mixture rich in tocotrienols, but the exact composition of the mixture was not clarified. Both treatments caused significant improvements in walking and standing visual analog scale and WOMAC score, but not waiting visual analog scale (Haflah et al., 2009). Vitamin E prevented the increase in malondialdehyde due to the progression of osteoarthritis, but glucosamine sulfate did not (Haflah et al., 2009). Only palm vitamin E increased the alpha-tocotrienol level in the blood of the patients (Haflah et al., 2009). However, the absence of a placebo arm in this trial is a major limitation. Osteoarthritis is an episodic disease, whereby the patients experience alternating episodes of symptoms and remission. Therefore, it is difficult to conclude that the treatments are beneficial without the placebo arm as the patients might be in remission.

A summary of the studies showing a positive relationship between vitamin E and joint health is presented in **Table 1**.

**Table 1:** Positive relationship between vitamin E and joint health.

Study type	Researchers (years)	Study design and subject characteristics	Findings	Notes
CC	Jordan et al., 2004	African–American and White adults from the Johnston County Osteoarthritis Project. 200 radiographic knee OA patients (KL ≥ 2) and 200 matched control (KL = 0) aged 45–92 years (mean 62.4 years). Tocopherols (TFs) were assayed using HPLC.	The OA patients had lower α:γTF ratio, higher γTF and higher δTF. Knee OA was negatively associated with serum αTF in men (OR: 0.1, CI: 0.01, 1.3), positively with serum γTF in men (OR: 21.8, CI: 1.8, 257), and not associated with serum δTF. Subjects at the highest tertile of α:γTF ratio had reduced risk for OA (adjusted odds ratio = 0.5, 95% CI: 0.2, 1.2), this association only persisted in African American (OR: 0.1 95% CI: 0.01, 0.6) and in men (OR: 0.02 CI: 0.001, 0.4). All the association not significant in women.	Temporal relationship could not be studied. Only vitamin E measured. One-time sampling. Circulating level might not reflect synovial level.

CC	Surapaneni and Venkataramana, 2007	20 Indian OA patients and 20 healthy control. Age of subjects between 35–60 years.	↓ Erythrocytic glutathione and catalase, vitamin E, vitamin C and ↑ erythrocytic MDA, GST and GPX levels in OA patients.	The blood oxidative status might not reflect the synovial status. Small sample size.

CC	Sutipornpalangkul et al., 2009	32 primary OA patients going for total knee replacement aged 55–88 years (mean age 69). 10 injured knee joint patients going for arthroscopy aged 19–42 years (mean age 25 years). Synovial vitamin E measured using HPLC.	↓ Synovial vitamin E level but not serum vitamin E in the OA patients.	Injured joint in control group might consume vitamin E as well.

CC	Suantawee et al., 2013	35 OA patients (24F: 11M), mean age 69.2 ± years. Diagnosed with clinical and radiographic knee OA (KL grades 3–4). 35 healthy control (26F: 9M), mean age 68.6 ± 1.2 years	↑ Plasma level of MDA and nitrite, ↓ vitamin E, TEAC and FRAP in OA patients. Correlation between: Synovial MDA and nitrite: + Vitamin E and MDA: − MDA and TEAC: − Plasma and synovial MDA, vitamin E and TEAC: nil	Synovial fluid was not taken from healthy subjects, so comparison could not be made. The lack of associations between plasma and synovial markers showed that they were not reflective to each other.

CC	Angthong et al., 2013	23 patients (mean age, 66.7 ± 7.6 years) with primary knee OA were divided into two groups: Severe knee society score (KSS) ≤ 46, *n* = 9. Mild-moderate KSS > 46, *n* = 14. αTF determined using HPLC.	↓ vitamin E level in severe OA group compared to mild-moderate group. Inverse correlation between KSS and vitamin E level.	Small sample size. No multivariate adjustment for confounding factors.

CS	Seki et al., 2010	562 Japanese subjects (224 male, 338 female) ≥ 40 years from Comprehensive Health. Examination Program. They were divided into OA (KL grade ≥ 2) or non-OA. TF isomer levels were determined using HPLC.	↑ β/γTF in non-OA subjects. In the logistic regression, risk of OA: Highest vs. lowest tertiles of β/γTF: OR: 0.52, 95% CI: 0.29–0.93. Middle vs. lowest tertiles of αTF: OR 0.51, 95% CI: 0.29–0.90.	The association between αTF and OA might be U-shaped. Subjects participating may be more health conscious than the general population.

CS	Muraki et al., 2014	827 Japanese rural participants (305 men and 522 women) aged > 40 years old (mean age: 69.2 ± 9.3 years). Self-administered brief diet history questionnaire. Minimum joint space width (mJSW) and osteophyte area (OPA) of the knee analysed using a computer-aided system.	Men: dietary nutrients were not associated with mJSW and OPA. Women: OPA was associated with vitamin E (*b* = −0.15, CI: −0.29 to −0.008).	Subjects might suffer from recall bias. The study might not account for changes in dietary habit across time although 90.4% of the subjects indicated that they had not changed their diet. The computer-grading system transformed KL grade system to continuous variable, which could provide higher strength in analysis.

CS	Barker et al., 2014a	56 subjects aged, 48 ± 1 years who were moderately active. Non-supplement users, non-NSAID users. They were classified based on vitamin D status: <20 ng/mL: deficient. 21–29 ng/mL: insufficient. >30 ng/mL: sufficient.	↑ γTF in vitamin D deficient subjects. In subjects with sufficient vitamin D, γTF showed significant inverse relationship with TNFα, which was associated with inflammation related quadricep muscle weakness.	Cross-sectional design and small sample size.

PS	McAlindon et al., 1996	640 subjects from Framingham Osteoarthritis Cohort Study. Incident OA: from KL ≤ 1 to KL ≥ 2. Progressive OA: KL increase by ≥ 1 on follow up. Baseline: 1983–1985 Follow up: 1992–1993 Intake of nutrients determined by self-administered food frequency questionnaire.	Incident OA was not associated with any nutrients. Vitamin E (middle vs. lowest tertile) was associated with progressive OA: OR 0.44, CI 0.19–1.00. The association was retained in men (highest vs. lowest tertile of vitamin E): OR 0.07 CI: 0.01–0.61.	Changes of dietary intake might occur with disease progression.

PS	Wang et al., 2016	214 participants aged 27–75 years (mean 67.4 years) from Melbourne Collaborative Cohort Study without diagnosed hip osteoarthritis who underwent hip magnetic resonance imaging in 2009–2010. 55.6% were women. Dietary intake measured by food frequency questionnaire using Australian food composition data. MRI scan performed on their dominant hip.	Higher intake of vitamin E (OR 0.63, CI 0.41–0.96) was associated with a reduced prevalence of femoral head cartilage defects but not with femoral neck bone marrow lesion.	MRI showed early changes that might be associated with OA before the occurrence of symptoms. Single dietary data were taken 20 years before the outcomes, the diet might change during this period. Subjects were relatively healthy.

CT	Haflah et al., 2009	Primary OA patients with KL grades 2–3, aged 58–59 years old, mix Malay and Chinese subjects. Control (*n* = 31): 1.5 g oral glucosamine sulphate. Treatment (*n* = 33): 400 mg oral palm vitamin E. Period: 6 months. Serum vitamin E determined by HPLC.	Both treatment improved walking, standing VAS and WOMAC score, but not waiting VAS. Vitamin E prevented the increase of MDA, but glucosamine did not. Palm vitamin E increased the serum α-tocotrienol level, but glucosamine did not.	X sample size calculation. X randomization. X blinding. √ dropout. Open labelled design. Exact composition of palm vitamin E mixture not indicated. No radiographic imaging of the joint.

CT	Bhattacharya et al., 2012	Healthy Indian individuals (control group, *n* = 40) and osteoarthritic patients aged 50–70 years supplemented with vitamin E 200 mg/day vitamin E (*n* = 40) for 3 months. All OA patients had KL grade 3.	Compared to normal control, OA patients had lower VAS, SOD, GPX, catalase activity in the blood, but higher ceruloplasmin, MDA, CRP, and synovial IL-6. After treatment, SOD, GPX, catalase activity increased significantly, while ceruloplasmin, MDA, levels decreased significantly. All analytes measured in the blood.	X sample size calculation. X randomization. X blinding. NA dropout. The circulating analytes might not reflect their level in the joint.

CT	Oikonomidis et al., 2014	46 patients diagnosed with osteoarthritis (OA) in one or both knees. Treatment: ascorbic acid 1 g two times daily plus vitamin E 100 mg three times daily. Control: meloxicam tabs 15m g 1 tab daily per os. Treatment period 20 days.	Vitamin C + E and meloxicam improved WOMAC and PVAS score but there is not significant difference between the groups. Vitamin C + E improved both extension and flexion deficits in mild-moderate and severe-extreme OA patients. Meloxicam only improved flexion deficit in severe-extreme patients. Vitamin C + E increased total antioxidant capacity of the synovial fluid better than meloxicam.	X sample size calculation. √ randomization. √ blinding. √ dropout. Small single-centre study.

CT	Tantavisut et al., 2017	Patients scheduled for total knee arthroplasty, KL grades 3–4. Treatment: 69.2 ± 1.3 years. Placebo: 69.5 ± 1.3 years. Treatment: Oral 400 IU of vitamin E for 2 months. Placebo control present. No NSAIDs, just paracetamol.	Vitamin E reduced MDA, increased αTF and Trolox equivalent antioxidant capacity in synovial fluid and blood. Vitamin E improved all domains of WOMAC scores (pain, stiffness, function) and Knee Society Score post-treatment. WOMAC score was inversely correlated with TEAC value. ↓ synovial tissue cells stained with nitrotyrosine and ↓ inflammatory cells in vitamin E group.	√ sample size calculation. √ randomization. √ blinding. √ dropout. Could be performed in patients with early OA. Authors think that vitamin E may have analgesic effects by suppressing protein kinase C and nitric oxide.

*CAT, catalase; CC, case-control study; CI, 95% confidence interval; CS, cross-sectional study; CT, clinical trial; FFQ, food frequency questionnaire; FRAP, ferric reducing antioxidant power; GPX, glutathione peroxidase; GST, glutathione transferase; HPLC, high-performance liquid chromatography; IL, interleukin; KL, Kellgren–Lawrence; MDA, malondialdehyde; MRI, magnetic resonance imaging; NSAID, non-steroidal anti-inflammatory drugs; OA, osteoarthritis; OR, odds ratio; PS, prospective study; SOD, superoxide dismutase; TEAC, Trolox equivalent antioxidant capacity; TF, tocopherol; TNF, tumor necrosis factor alpha; WOMAC, Western Ontario and McMaster Universities Arthritis Index; VAS, visual analog scale; X, not mentioned; √, mentioned.*

### Negligible Relationship Between Vitamin E and Joint Health

A case-control study among advanced (Kellgren–Lawrence scale 3–4, WOMAC pain score > 2, muscle weakness; mean age: 43 ± 12 years) and early (only presented with 1–2 criteria listed earlier; mean age 42 ± 11 years) osteoarthritic patients by Barker et al. (2014b) indicated that despite elevated systemic inflammation markers (tumor necrosis factor alpha, interleukin-5, −15, −12, and −13) in advanced patients, the micronutrient levels including vitamin E were similar between patients at different stages of osteoarthritis (Barker et al., 2014b). It could not be ruled out that antioxidant like vitamin E had been depleted in the early stage of osteoarthritis, thus obliviating the differences between patients of difference stages. There might also be a difference between circulating and synovial level of vitamin E, which the latter would be more relevant in osteoarthritic patients.

In a large cross-sectional study involving 4685 Chinese subjects aged 40–85 years, vitamin C but not vitamin E intake was associated with radiographic knee osteoarthritis after adjustment for confounding variables (Li et al., 2016). The authors suggested that vitamin C, a hydrophilic compound, penetrated joint space better than vitamin E, a lipophilic compound. In addition, intake level assessed through food frequency questionnaire might not reflect the level of nutrients in the blood/joint space. In the Melbourne Collaborative Cohort Study, Wang et al. (2007) followed 293 healthy adults (mean age at baseline 58.0 ± 5.5 years) for 10 years (Wang et al., 2007). Osteoarthritic changes at the knee, defined as cartilage volume, bone area, cartilage defects and bone marrow lesions, were assessed by magnetic resonance imaging. The study demonstrated a reduced risk for bone marrow lesions (OR: 0.50, 95% CI: 0.29–0.87), reduction in tibial plateau bone area (β = −35.5, 95% CI: −68.8 to −2.3) in subjects with higher vitamin C intake (Wang et al., 2007). Those with higher vitamin E intake seemed to have a higher tibial plateau bone area but it was not statistically significant (β = 33.7, 95% CI = −3.1 to 70.4) (Wang et al., 2007). The authors acknowledged that dietary record was taken 10 years prior to outcomes measurement, thus changes might be expected over time. Moreover, intake of supplements was not considered in this study.

Two trials showed that vitamin E supplementation alone was not effective in retarding functional and biological degeneration of the knee in osteoarthritic patients. Brand et al. (2001) treated 72 Australian patients with radiographic knee osteoarthritis with either 500 IU vitamin E (mean age = 67.1 ± 1.4 years) or placebo (mean age = 66.1 ± 1.5 years) for 6 months (Brand et al., 2001). No significant improvements in pain, stiffness, function, frequency of pain, category of pain and observer global assessment were observed between groups and across time (Brand et al., 2001). In the subsequent study, Wluka et al. (2002) supplemented Australian osteoarthritic patients with 500 IU vitamin E (*n* = 59, mean age = 64.3 ± 11 years) or placebo (*n* = 58, mean age = 63.7 ± 10 years) for 2 years and their knee was scanned using magnetic resonance imaging (Wluka et al., 2002). The study showed that changes in knee cartilage volume, WOMAC and SF-36 scores were similar between the supplemented and the placebo groups (Wluka et al., 2002).

Two clinical trials demonstrated that the combination of vitamin with other agents did not improve the disease progression in osteoarthritic patients. Aydogan et al. (2008) showed that the combination of oral vitamin E (400 IU/day) and intraarticular Hylan G-F 20 (a derivative of hyaluronan) injection did not improve the circulating and synovial catalase, superoxide dismutase and glutathione peroxidase activity, as well as malondialdehyde level in patients with knee pain and osteoarthritis (aged 40–68 years) compared to patients undergoing arthroscopy, intraarticular Hylan G-F 20 or Na-hyaluronate injection (Aydogan et al., 2008). Medhi et al. (2011) determined the efficacy of vitamin E (200 IU/day) and vitamin C (500 IU/day) co-supplementation on patients with primary osteoarthritis given paracetamol (1 g/twice daily) (Medhi et al., 2011). Both the supplemented (*n* = 50; mean age = 54.84 ± 10.64 years) and unsupplemented group (*n* = 50, mean age = 54.84 ± 10.64 years) experienced similar improvement in pain assessed via visual analog scale throughout the 8 weeks period, and the effect of supplementation was not significant (Medhi et al., 2011).

### Negative Relationship Between Vitamin E and Joint Health

As mentioned earlier, Johnston County Osteoarthritis Project case-control sub-study demonstrated that gamma-tocopherol was positively associated with osteoarthritis in men (OR: 21.8, 95% CI: highest tertile: 1.8–257; highest tertile versus lowest tertile) (Jordan et al., 2004). Subjects with the highest ratio of alpha- to gamma-tocopherol were associated with reduced risk of osteoarthritis (OR: 0.5, 95% CI: 0.2–1.2; versus the lowest tertile) (Jordan et al., 2004). This relationship was persistent in African American subjects (OR: 0.1, 95% CI: 0.01, 0.6) and in men (OR: 0.02 CI: 0.001, 0.4) (Jordan et al., 2004). Delta-tocopherol was not associated with osteoarthritis in this population (Jordan et al., 2004). Despite the large sample size, this study only measured the circulating vitamin E levels due to the invasive nature of synovial sampling, thus it might not reflect the synovial level. In the prospective case-control study by Chaganti et al. (2014), 145 osteoarthritic patients (mean age = 61.7 ± 7.7 years) and 282 healthy controls (mean age = 61.5 ± 7.7 years) were followed up for 30 months (Chaganti et al., 2014). They demonstrated that high circulating vitamin C (OR: 2.20, 95% CI: 1.12–4.33, highest vs. lowest tertile) and vitamin E (alpha-tocopherol; OR 1.89, 95% CI 1.02–3.50, highest versus lowest tertile) were associated with a high incident of whole knee OA (Chaganti et al., 2014). Since patients in the early stage of the disease might take supplements, a subanalysis was performed on non-users but the observation remained (Chaganti et al., 2014). It was suggested that high level of antioxidants like vitamin C and vitamin E might act as a prooxidant and harmed the cartilage. This was advocated by the study of Seki et al. (2010), whereby subjects at the middle tertile of alpha-tocopherol was associated with lower risk for osteoarthritis in Japanese subjects (OR 0.51, 95% CI: 0.29–0.90) but not the highest tertile (Seki et al., 2010). These observations suggest a U-shaped relationship between vitamin E and joint health – beneficial at lower concentrations, harmful at higher concentrations. The prooxidant activity of vitamin E has been demonstrated *in vitro* and *in vivo* by other researchers (Tafazoli et al., 2005; Pearson et al., 2006), so high-dose vitamin E in patients is cautioned.

A summary of the studies showing a negligible or negative relationship between vitamin E and joint health is presented in **Table 2**.

**Table 2:** Negligible or negative relationship between vitamin E and joint health.

Study type	Researchers (years)	Study design and subject characteristics	Findings	Notes
CC	Barker et al., 2014b	Advanced OA: pain > 2 on WOMAC, muscle weakness (measured by isokinetic knee-extension or flexion torque), KL grades 3–4. *n* = 14; females = 8; age, 43 ± 12 years; Early OA: 1–2 of the above. *n* = 14; females = 8; age, 42 ± 11 years. αTF measured using HPLC.	↑ TNFα, IL-5, IL-12, and IL-13 in the early OA group. Vitamin E level was similar between the two groups.	Small sample size.

CS	Li et al., 2016	4685 Chinese participants > 40–85 years old undergoing regular health check-up. Dietary intake: semi-quantitative food frequency questionnaire (FFQ). Radiographic knee OA was defined as K-L grade 2.	Only vitamin C was associated with knee radiographic OA after multiple adjustments. Other antioxidants, such as carotenoids, vitamin E and selenium, were not significantly related with OA.	FFQ records may not reflect the level of nutrients in the blood and in the joint. Large sample size.

PS	Wang et al., 2007	293 healthy adults (mean age = 58.0 ± 5.5 years) without knee pain or injury from Melbourne. Collaborative Cohort Study. Intake of vitamins from FFQ. Cartilage volume, bone area, cartilage defects and bone marrow lesions defined using MRI after 10 years.	↑ vitamin E intake, ↑ tibial plateau bone area (beta = 33.7, 95% CI = −3.1 to 70.4, *P* = 0.07)	Diet intake might change over time. Intake of supplements not considered.

PS CC	Chaganti et al., 2014	145 cases aged 61.7 ± 7.7 years and 282 controls aged 61.5 ± 7.7 years. They were followed up for 30 months. Plasma vitamin C and serum vitamin E (αTF) were measured using HPLC.	Higher vitamin C levels [highest tertile vs. lowest tertile, adjusted OR: 2.20 (CI: 1.12–4.33) and vitamin E (highest tertile vs. lowest tertile, OR: 1.89 (1.02–3.50)] were associated with high incidence of whole knee OA.	Single measurement of vitamin C and vitamin E. Vitamin E level was not adjusted with cholesterol. Circulating level is better measurement than FFQ.

CT	Brand et al., 2001	72 Subjects from Australia with radiographic OA and pain. They were randomized into treatment group receiving 500 IU (age 67.1 ± 1.4 years) or placebo (age 66.1 ± 1.5 years) for 6 months.	The two groups were not significantly different between pain, stiffness, function, frequency of pain, categorical pain, and observer global assessment (between group and across time).	X sample size calculation. √ randomization. √ blinding. √ dropout. The baseline pain level in the placebo group was higher. No measurement of biochemical markers.

CT	Wluka et al., 2002	Subjects from Australia with radiographic OA and pain. 136 patients with knee OA were randomized into treatment group receiving 500 IU (*n* = 59, age 64.3 ± 11 years) or placebo (*n* = 58, age 63.7 ± 10 years) for 2 years. Tibial cartilage volume was measured using MRI.	Changes in knee cartilage volume was similar between the treatment and placebo groups before or after multiple adjustments. WOMAC and SF-36 were similar between two groups. Overall, intake of vitamin C, beta-carotene, and retinol equivalents were not associated with changes in knee cartilage volume.	X sample size calculation. √ randomization. X blinding. √ dropout.

CT	Aydogan et al., 2008	Age of the patients was 52.53 years (40–68 years) 60 patients with knee pain and OA were randomized equally into four groups: (1) Intraarticular Hylan G-F 20 for 3 weeks. (2) Intraarticular Hylan G-F 20 + oral vitamin E 400 IU/day for 3 weeks. (3) Arthroscopy. (4) Intraarticular Na-hyaluronate for 5 weeks.	Blood CAT, MDA, SOD, and GPX did not differ among the groups. Synovial MDA was different between groups but pairwise comparison not performed. Synovial SOD and GPX did not differ among the groups.	X sample size calculation. X randomization. X blinding. No dropout. Small sample size. No functional measurement.

CT	Medhi et al., 2011	Patients with primary OA. Control (mean age 52.80 ± 9.20 years): paracetamol 1 g (*n* = 50) twice daily for 8 weeks. Treatment (mean age 54.84 ± 10.64 years): paracetamol 1 g twice daily with vitamin C (500 mg) and E (200 IU) (*n* = 50).	Based on VAS, both group showed improvement and there was no significant difference between these two groups during 2nd (2 weeks) and 3rd visit (4 weeks). After 8th week (last visit), the antioxidant treated group performed better according to the authors.	X sample size calculation. X randomization. X blinding. √ dropout. Single outcome measured.

CT	Dehghan, 2015	120 Iranian patients aged 30–60 years diagnosed with osteoarthritis. Three groups: control, vitamin B, and vitamin E (alpha-tocopherol 100 mg). All the groups took diclofenac 50 mg twice a day.	Across time, improvement in pain VAS, past 48 h function test, total pain severity was significantly higher in vitamin B compared to vitamin E and control group. At 2nd visit (14th day), the past 48 h function tend to be better in vitamin E compared to vitamin B and placebo. During the 3rd visit (21st day), all WOMAC subscales were better in vitamin B group than vitamin C but not significantly different.	X sample size calculation. X randomization. X blinding. √ dropout. Dose of vitamin E used was low compared to other studies. Short follow up period.

*CAT, catalase; CC, case-control study; CI, 95% confidence interval; CS, cross-sectional study; CT, clinical trial; FFQ, food frequency questionnaire; GPX, glutathione peroxidase; HPLC, high-performance liquid chromatography; IL, interleukin; KL, Kellgren–Lawrence; MDA, malondialdehyde; MRI, magnetic resonance imaging; OA, osteoarthritis; OR, odds ratio; PS, prospective study; SOD, superoxide dismutase; TF, tocopherol; TNF, tumor necrosis factor alpha; WOMAC, Western Ontario and McMaster Universities Arthritis Index; VAS, visual analog scale; X, not mentioned; √, mentioned.*

## Perspectives

In most of the previous studies, the term vitamin E is used loosely to refer to alpha-tocopherol. This might stem from the fact that alpha-tocopherol is most abundant in nature and the recommended nutrient intake of vitamin E is based on alpha-tocopherol (Institute of Medicine (Us) Panel on Dietary Antioxidants and Related Compounds, 2000). This might not be appropriate because vitamin E is a vast family consisting of 8 distinct isomers belonging to two major groups, which possess distinct biological activities. As illustrated in the study by Jordan et al. (2004), even members of the same vitamin E family exerted different effects on the progression of osteoarthritis, whereby alpha-tocopherol was beneficial but gamma-tocopherol was detrimental to the cartilage (Jordan et al., 2004). Although causality cannot be derived from this observational study, it did provide a hypothesis that different vitamin E isomers had different effects on the joints. The researchers should be specific when referring to which isomers they were testing in their studies.

Most of the trials supplemented patients with osteoarthritis with alpha-tocopherol and only one study used palm vitamin E mixture rich in tocotrienols (Haflah et al., 2009). Currently, there is no study comparing the efficiency of alpha-tocopherol and tocotrienols. Tocotrienol possesses some biological activities not exhibited by alpha-tocopherol, for example, its effects in suppressing mevalonate pathway important in cholesterol synthesis, bone remodeling and carcinogenesis (Mo et al., 2012; Shen et al., 2017). In addition, previous studies showed that tocotrienols exerted superior antioxidant, anti-inflammatory, and antiosteoporotic activities compared to alpha-tocopherol (Ahmad et al., 2005; Norazlina et al., 2007; Maniam et al., 2008). Therefore, it is reasonable to postulate that tocotrienol might be more effective than alpha-tocopherol in treating osteoarthritis.

However, the development of tocotrienol as an alternative supplement to alpha-tocopherol is hindered by the low bioavailability of tocotrienol in the body (Fu et al., 2014). This is due to the presence of alpha-tocopherol transfer protein in the liver, which preferentially binds and traffics alpha-tocopherol into the circulation (Hosomi et al., 1997). In addition, the joint space is an aqueous environment and the cartilage layer is avascular. Hence, it is a challenge to deliver hydrophobic substances to the joint. The bioavailability of vitamin E in the joint space has not been studied. More effective approach to deliver it to the joint space, such as interarticular injection and the use of structurally modified vitamin E which are more hydrophilic, should be tested.

Osteoarthritis is a disease involving the cartilage, subchondral bone, tendon, synovium and muscles. Joint instability due to muscle and bone can cause unequal mechanical loading of the joint, contributing to osteoarthritis (Egloff et al., 2012). Early stage of osteoarthritis was associated with increased bone resorption, followed by increased bone formation at the later stage (Li et al., 2013). Previous studies showed that vitamin E, in the form of alpha-tocopherol, individual tocotrienols or mixture of both exerted bone-sparing effects in various animal models of bone loss (Chin and Ima-Nirwana, 2012, 2015). The effects of high-dose alpha-tocopherol on the skeleton are debatable because both adverse and negligible effects have been reported (Fujita et al., 2012; Iwaniec et al., 2013). Vitamin E was also reported to prevent muscle weakness and sarcopenia in preclinical models (Khor et al., 2014). The effects of vitamin E on the bone and muscle were not discussed in detail in this review. However, future studies should consider the pleiotropic effects of vitamin E in explaining its action in relieving osteoarthritis.

Most of the current studies attributed the chondroprotective effects of vitamin E to its antioxidative effects. However, vitamin E can also influence other molecular signaling involved in the cartilage remodeling and chondrocyte survival. For instance, transforming growth factor beta is required for the development of articular cartilage but at high concentration may cause cartilage degradation in adults (Li and Xu, 2015). Vitamin E was reported to inhibit transforming growth factor beta signaling in various cell types. Sirtuin-1 has been implicated in the senescence of chondrocytes and pathogenesis of osteoarthritis. A recent study showed that the combination of vitamin E (400 IU/day) and omega-3 fatty acid (4 g/day) for 2 months upregulated sirtuin-1 gene expression of the peripheral blood mononuclear cells in patients with coronary artery disease (Saboori et al., 2016). However, the involvement of sirtuin-1 and transforming growth factor beta in the cartilage-sparing effects of vitamin E has not been validated yet.

Previous studies on vitamin E supplementation suggested that baseline antioxidant level or redox status of the patients should be considered. A study among the obese subjects showed that vitamin E caused a greater reduction in plasma F_2_-isoprostanes (from −3.9% to −9.8%) when the baseline F_2_-isoprostanes was higher than 50 μg/mL (Block et al., 2008). In this context, the influence of baseline antioxidant level or redox status on the chondroprotective effects of vitamin E should be investigated. This is feasible through assessment of stable *in vivo* oxidative stress markers like urinary levels of 8-iso-PGF2a before initiating supplementation (Patrignani et al., 2000).

## Conclusion

Oxidative stress is one of the underlying mechanisms contributing to cartilage degeneration in osteoarthritis. This is evidenced by the reduced antioxidant (including vitamin E) and increased lipid peroxidation products in the circulation and synovial fluid of the patients with osteoarthritis. The association between vitamin E level and the induction/progression of osteoarthritis in the general populations remains debatable due to the heterogenous findings in observational studies. The effects of vitamin E supplementation in retarding the progression of osteoarthritis in patients is still debatable due to the heterogeneous outcomes. High-dose vitamin E supplementation is cautioned due to its potential prooxidant effects. Different isoforms of vitamin E may have distinct biological effects on joint health but the studies on isoforms others than alpha-tocopherol are limited. This is a major research gap that should be addressed in future studies to validate the use of vitamin E in tackling osteoarthritis.

## Author Contributions

K-YC and SI-N contributed equally to the writing of this review. K-YC and SI-N agreed to be accountable for the content of the work.

## Conflict of Interest Statement

The authors declare that the research was conducted in the absence of any commercial or financial relationships that could be construed as a potential conflict of interest.
